# Design of a Multi-Position Alignment Scheme

**DOI:** 10.3390/s24061938

**Published:** 2024-03-18

**Authors:** Bofan Guan, Zhongping Liu, Dong Wei, Qiangwen Fu

**Affiliations:** 1 School of Automation, Northwestern Polytechnical University, Xi’an 710072, China; gbf1016@126.com (B.G.); weid@mail.nwpu.edu.cn (D.W.); 2 Shaanxi Aircraft Corporation, Xi’an 723214, China; liuzpwy@163.com

**Keywords:** inertial navigation system (INS), error modulation, alignment, scale factor error

## Abstract

The current new type of inertial navigation system, including rotating inertial navigation systems and three-autonomy inertial navigation systems, has been increasingly widely applied. Benefited by the rotating mechanisms of these inertial navigation systems, alignment accuracy can be significantly enhanced by implementing IMU (Inertial Measurement Unit) rotation during the alignment process. The principle of suppressing initial alignment errors using rotational modulation technology was investigated, and the impact of various component error terms on alignment accuracy of IMU during rotation was analyzed. A corresponding error suppression scheme was designed to overcome the shortcoming of the significant scale factor error of fiber optic gyroscopes, and the research content of this paper is validated through corresponding simulations and experiments. The results indicate that the designed alignment scheme can effectively suppress the gyro scale factor error introduced by angular motion and improve alignment accuracy.

## 1. Introduction

Initial alignment is one of the key technologies in inertial navigation [1,2], and the inertial navigation system (INS) needs to perform initial alignment before entering the navigation state during every startup. Due to the fact that inertial navigation calculation is an integral process, and initial alignment is actually a problem of determining the initial value of integration [3], the alignment result will directly affect the navigation accuracy of the system [4]. Therefore, INS requires accuracy for initial alignment. It requires reducing the initial alignment error to introduce irreversible effects on navigation solutions.

At present, with the application of new INSs such as rotating INS [5,6,7,8] and three-autonomy (autonomous calibration, autonomous alignment and autonomous test) INS [9], some new alignment schemes have also emerged. Due to the fact that the above-mentioned INS has a rotating mechanism, schemes such as multi-position alignment and rotation alignment have been proposed [10,11].

The multi-position alignment scheme utilizes the error estimation and compensation principle of IMU to improve alignment accuracy [12]. The two-position alignment scheme, which rotates 180° around the azimuth axis, is a widely applied multi-position alignment scheme. Fang et al. [13] addressed the issue of poor accuracy in high-precision airborne POS ground two-position alignment caused by output noise from inertial devices by using an adaptive Kalman filter to improve alignment accuracy. Lai et al. [14] proposed an arithmetic model of a fast two-position initial alignment for INS using unscented Kalman filter. Zhang et al. [15] used a piecewise combined Kalman filter utilizing relative azimuth constraint to improve the alignment precision and to reduce the time consumption for error convergence.

Compared with multi-position alignment, rotation alignment is another approach, which uses the principle of error modulation to suppress the impact of IMU-related errors on alignment accuracy through the periodic rotation of the IMU. Wang and Li [16] used a rotation operation and calculated the error based on the projection relationship of the alignment steady-state error at different positions. By compensating for the error, the alignment accuracy of the system is improved. Wei et al. [17] designed a new initial alignment algorithm for dual-axis rotation alignment to solve the problem of azimuth error divergence caused by traditional alignment algorithms when IMU rotates around the horizontal axis.

Because the additional angular motion is introduced by the above alignment methods, analyses of the impact of inertial component errors on the system under rotational conditions term by term are necessary. In this study, detailed analyses of various gyroscope errors under angular motion were conducted, and the impact of these errors on alignment accuracy was assessed. Owing to the coupling of rotation and gyro scale factor error during the alignment process, new additional errors are introduced. Under the condition of large-scale factor errors in gyroscopes, existing two-position alignment schemes will produce significant errors that cannot be ignored. A new multi-position alignment scheme was designed based on the two-position alignment scheme, which effectively suppresses the impact of gyro scale factor error.

The remainder of this paper is structured as follows: The Section 2 elucidates the coordinate definitions employed in the analysis and delineates the error modes inherent to INS. Subsequently, the Section 3 concentrates on scrutinizing the influence of gyroscope errors on the alignment process under rotational circumstances, and devises a multi-position alignment methodology predicated on these analyses. This is followed by the Section 4, wherein the efficacy of the designed multi-position alignment scheme is empirically validated through mathematical simulations and experimental investigations. Finally, the conclusions drawn from this study are encapsulated in the Section 5.

## 2. Coordinate System and Error Model of INS

### 2.1. Coordinate System Definition

In the strapdown inertial navigation system, the commonly used coordinate systems include inertial frame, earth frame, geographic frame, navigation frame, body frame, etc. In the rotation inertial navigation system, another IMU frame is added [18,19]. The coordinate system used in the subsequent analysis of this paper is defined as follows:

Inertial frame (*i* frame)—$O_{e}x_{i}y_{i}z_{i}$: The origin is located in the center of the earth, and the coordinate axis has no rotation relative to the star. Axis $O_{e}z_{i}$ is traditionally selected along the north pole of the earth. Axis $O_{e}x_{i}$ and axis $O_{e}y_{i}$ are selected in the equatorial plane of the earth and point to two stars in space.

Earth frame (*e* frame)—$O_{e}x_{e}y_{e}z_{e}$: The origin is at the center of the earth, and the coordinate axis is firmly connected with the earth. Axis $O_{e}z_{e}$ points to the direction of the earth’s polar axis. Axis $O_{e}x_{e}$ is along the intersection of the Greenwich meridian and the equatorial plane of the earth. The earth frame rotates around axis $O_{e}z_{i}$ at angular velocity $\omega_{ie}$ relative to the inertial frame.

Geographic frame (*g* frame)—$Ox_{g}y_{g}z_{g}$: The coordinate origin is located at the center of gravity of the carrier. Axes $ox_{g}$ and $oy_{g}$ are at the local level, where axis $ox_{g}$ points east and axis $oy_{g}$ points north. Axis $oz_{g}$ points to the sky along the vertical direction.

Navigation frame (*n* frame)—$ox_{n}y_{n}z_{n}$: The navigation frame is selected as the navigation reference according to the needs of the system during navigation. This paper uses the geographic frame as the navigation frame.

Body frame (*b* frame)—$ox_{b}y_{b}z_{b}$: The body frame is fixed on the carrier. The coordinate origin *o* of the body frame is located at the center of gravity of the carrier. Axis $ox_{b}$ points to the right along the horizontal axis of the carrier, and axis $oy_{b}$ points forward along the longitudinal axis of the carrier. Axes $oz_{b}$, $ox_{b}$ and $oy_{b}$ form a right-handed coordinate system.

IMU frame (*p* frame)—$ox_{p}y_{p}z_{p}$: The coordinate origin of the IMU frame coincides with that of the body frame, which is a unique frame of the rotating inertial navigation system. Axes are the directions of the sensitive axes of the inertial components. When each rotation angle of the shaft is zero, the *p* frame coincides with the *b* frame.

### 2.2. Error Model of IMU

The error models for the gyroscope and accelerometer are shown in Equations (1) and (2) [20]:

(1) $$\delta \omega_{ip}^{p}=\left(\delta K_{g}+G\right)\omega_{ip}^{p}+\varepsilon$$

(2) $$\delta f_{ip}^{p}=\left(\delta K_{a}+A\right)f_{ip}^{p}+\nabla$$

where $\delta \omega_{ip}^{p}={\left[\begin{array}{ccc} \delta \omega_{ipx}^{p} & \delta \omega_{ipy}^{p} & \delta \omega_{ipz}^{p} \end{array}\right]}^{T}$ and $\delta f_{ip}^{p}=\left[\begin{array}{ccc} \delta f_{ipx}^{p} & \delta f_{ipy}^{p} & \delta f_{ipz}^{p} \end{array}\right]$ represent the output error of the gyro and accelerometer, respectively; $\omega_{ip}^{p}={\left[\begin{array}{ccc} \omega_{ipx}^{p} & \omega_{ipy}^{p} & \omega_{ipz}^{p} \end{array}\right]}^{T}$ and $f_{ip}^{p}=\left[\begin{array}{ccc} f_{ipx}^{p} & f_{ipy}^{p} & f_{ipz}^{p} \end{array}\right]$ respectively represent angular velocity and acceleration input. The superscript *p* indicates that the measurement coordinate system is the *p* frame, the subscript *ip* indicates the angular velocity or acceleration of the *p* frame relative to the *i* frame, and x, y, z represent the corresponding coordinate axes. $\varepsilon ={\left[\begin{array}{ccc} \varepsilon_{x} & \varepsilon_{y} & \varepsilon_{z} \end{array}\right]}^{T}$ and $\nabla ={\left[\begin{array}{ccc} \nabla_{x} & \nabla_{y} & \nabla_{z} \end{array}\right]}^{T}$ represent the constant drift of gyro and the constant bias of accelerometer respectively. $\delta K_{g}=diag\left({\left[\begin{array}{ccc} \delta K_{gx} & \delta K_{gy} & \delta K_{gz} \end{array}\right]}^{T}\right)$ and $\delta K_{a}=diag\left({\left[\begin{array}{ccc} \delta K_{ax} & \delta K_{ay} & \delta K_{az} \end{array}\right]}^{T}\right)$ respectively represent the scale factor error of the gyro and accelerometer. Gyro misalignment angle **G** and accelerometer misalignment angle **A** represent the included angle between the IMU-sensitive axis and the *p* frame, which could be expressed as:

(3) $$G=\left[\begin{array}{ccc} 0 & G_{xy} & G_{xz}\\ G_{yx} & 0 & G_{yz}\\ G_{zx} & G_{zy} & 0 \end{array}\right]$$

(4) $$A=\left[\begin{array}{ccc} 0 & A_{xy} & A_{xz}\\ A_{yx} & 0 & A_{yz}\\ A_{zx} & A_{zy} & 0 \end{array}\right]$$

### 2.3. Error Model of INS

The attitude error equation and velocity error equation of the INS are shown in Equations (5) and (6) [21]:

(5) $$\dot{\phi }=\phi \times \omega_{in}^{n}+\delta \omega_{in}^{n}-C_{p}^{n}\delta \omega_{ip}^{p}$$

(6) $$\delta {\dot{V}}^{n}=-\phi \times f^{n}+\delta V^{n}\times \left(2\omega_{ie}^{n}+\omega_{en}^{n}\right)+V^{n}\times \left(2\delta \omega_{ie}^{n}+\delta \omega_{en}^{n}\right)+C_{p}^{n}\delta f_{ip}^{p}$$

where $\phi ={\left[\begin{array}{ccc} \phi_{E} & \phi_{N} & \phi_{U} \end{array}\right]}^{T}$ is attitude error; $\delta V^{n}={\left[\begin{array}{ccc} \delta V_{E} & \delta V_{N} & \delta V_{U} \end{array}\right]}^{T}$ is velocity error; $\omega_{ie}^{n}$ is Earth’s rotational motion, $\omega_{en}^{n}$ is the rotation angular velocity of the *n* frame, and $\omega_{in}^{n}=\omega_{ie}^{n}+\omega_{en}^{n}$; $f^{n}={\left[\begin{array}{ccc} f_{E} & f_{N} & f_{U} \end{array}\right]}^{T}$ is the specific force output under the navigation frame; $V^{n}={\left[\begin{array}{ccc} V_{E} & V_{N} & V_{U} \end{array}\right]}^{T}$ indicates the speed of the carrier; and $C_{p}^{n}$ represents the transformation matrix from the *p* frame to the *n* frame.

## 3. Error Analysis and Scheme Design for Alignment

### 3.1. Error Analysis for Alignment

In general, the carrier remains stationary during the alignment process. Theoretically, the speed output of the system is zero, but the actual speed output of INS is not zero due to the inertial sensor error. Therefore, the speed output is used as the measurement to inform the alignment process [22]. During the alignment process, the alignment accuracy of the horizontal attitude of the system depends on the equivalent bias of the horizontal accelerometers $\nabla_{E}$ and $\nabla_{N}$, and the initial alignment accuracy of the azimuth depends on the equivalent bias of the east accelerometer $\nabla_{E}$ and the equivalent drift of the east gyro $\varepsilon_{E}$. Considering that gyro accuracy is the main factor affecting the alignment accuracy, this paper mainly analyzes the effect of gyro error term by term.

#### 3.1.1. Constant Bias

**Figure 1:**
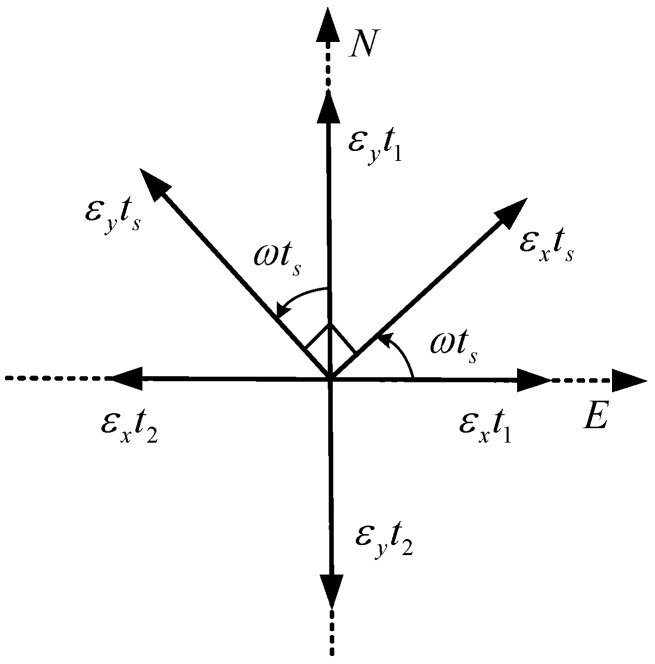
Rotation diagram of $\varepsilon_{x}$ and $\varepsilon_{y}$ during two-position alignment.

For the two-position scheme, during the alignment process, the IMU basically remains at the horizontal position, and remains stationary at the first position for a period of time. Then the IMU rotates 180° around the azimuth axis to reach the second position, and remains stationary for the same time. Assuming the initial attitude is [0°, 0°, 0°], when the coarse alignment error is ignored, the horizontal gyro bias before and after rotation is shown in Figure 1.

At the first stationary position, $\varepsilon_{xt_{1}}$ is the easterly component of the *x*-axis gyro, i.e., the equivalent easterly gyro drift ω presents the rotation angular rate of IMU. At the second stationary position, $\varepsilon_{xt_{2}}$ is the equivalent easterly gyro drift. During the rotation, the equivalent eastward gyro drift caused by the *x*-axis gyro is:

(7) $$\varepsilon_{xt_{s}}=\frac{\int_{0}^{t_{s}}\varepsilon_{x}\cos\left(\omega t\right)dt}{t_{s}}$$

Considering the rotation process and the two stationary positions, the equivalent eastward gyro drift caused by the *x*-axis gyro in the two-position alignment process is:

(8) $$\varepsilon_{xE}=\varepsilon_{xt_{1}}+\varepsilon_{xt_{s}}+\varepsilon_{xt_{2}}=\frac{\int_{0}^{t_{1}}\varepsilon_{x}dt+\int_{0}^{t_{s}}\varepsilon_{x}\cos\left(\omega t\right)dt-\int_{0}^{t_{2}}\varepsilon_{x}dt}{t}$$

In Equation (8), the integration of the constant drift of the gyroscope during the stationary position and rotation process can be understood as the accumulation of the effect of the gyro drift over a period of time. By adding the integrals of the three processes and averaging them to the whole alignment time, the average equivalent result of gyro drift in the east direction during the whole alignment process can be obtained. $\varepsilon_{xE}$ can be expressed as equivalent eastward gyro drift caused by *x*-axis gyro bias during two-position alignment. It can be seen from Equation (8) that when the IMU is stationary at two positions for the same time, the equivalent eastward gyro bias caused by $\varepsilon_{x}$ is zero.

We can then analyze $\varepsilon_{y}$ in the same way. Since the *y*-axis initially points to the north, the equivalent eastward gyro drift caused by the *y*-axis gyro in the two-position alignment process is the equivalent component in the rotation process, namely:

(9) $$\varepsilon_{yE}=\varepsilon_{yt_{s}}=-\frac{\int_{0}^{t_{s}}\varepsilon_{y}\sin\left(\omega t\right)dt}{t}\approx -\frac{2\varepsilon_{y}}{\omega t}$$

The estimation accuracy of azimuth error caused by horizontal gyro drift can be obtained by combining Equations (8) and (9):

(10) $$\delta {\hat{\phi }}_{U}^{\varepsilon }=-\frac{\varepsilon_{E}}{\omega_{ie}\cos L}=-\frac{2\varepsilon_{y}}{\omega t\omega_{ie}\cos L}$$

where $\delta {\hat{\phi }}_{U}^{\varepsilon }$ is the estimation accuracy of azimuth error, *L* is the local latitude, and $\omega_{ie}$ is the rotational velocity of earth.

It can be seen from Equation (10) that although the *y*-axis gyro bias will affect the alignment accuracy, its impact is very small. According to the above analysis process, it can be concluded that the effect of gyro constant bias on azimuth alignment accuracy can be well suppressed by IMU staying in the horizontal symmetric position at the same time.

**Figure 2:**
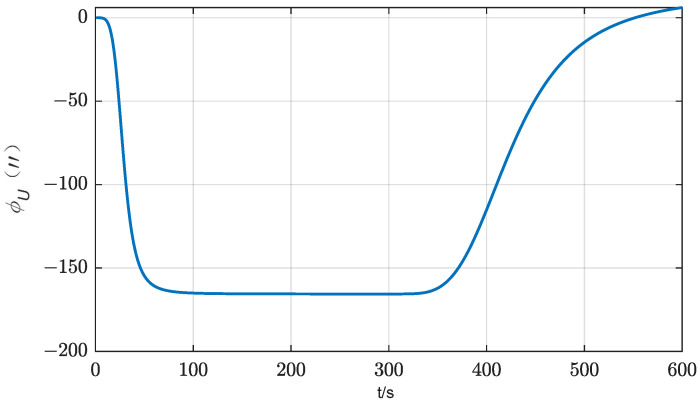
Influence of *x*-axis gyro bias on azimuth error (two-position scheme).

A simple simulation analysis of single item error for the above theoretical analysis is then carried out. IMU stands still for 295 s at the first position, then rotates 180° around the azimuth axis to the second position within 10 s and stands still for 295 s. Assume that the constant drift of horizontal axis gyro is 0.01°/h, and ignore other component errors and coarse alignment errors. The initial attitude is [0°, 0°, 0°]. The simulation results are shown in Figure 2.

No matter the alignment process of the first position or the second position, the effect of the *y*-axis gyro drift on the azimuth alignment is not significant, and from the alignment results, there is basically no significant difference. The reason is that the *y*-axis points to the north at both positions, and the component in the east direction is always small. The effect of the rotation process on the east component is relatively small from the average to the whole alignment period. However, as shown in Figure 2, the effect of *x*-axis gyro drift is quite remarkable. The azimuth error of the first position is relatively large, but the azimuth error of the second position is gradually reduced. When the alignment time of the two positions is consistent, the azimuth error is basically consistent with the situation without component error. It can be seen that the second position gradually modulates the azimuth error generated in the alignment process of the first position.

*Z*-axis gyro drift will also cause azimuth alignment error. When the rotation axis of two-position alignment basically coincides with the horizontal plane, in the first position, the azimuth drift introduced by $\varepsilon_{z}$ is $-\varepsilon_{z}t_{1}$, whose minimum variance estimation error is $-\varepsilon_{z}t_{1}/2$; similarly, in the second position, the minimum variance estimation error introduced by $\varepsilon_{z}$ is $-\varepsilon_{z}t_{2}/2$. In the whole two-position alignment process, the azimuth estimation error caused by $\varepsilon_{z}$ is:

(11) $$\delta {\hat{\phi }}_{U}^{\varepsilon_{z}}=-\frac{\varepsilon_{z}t}{2}$$

The azimuth estimation error caused by the *z*-axis gyroscope with constant drift of 0.01°/h used in the later analysis in the 10-min alignment time is about 6″.

For the rotation scheme, IMU rotates in full circle around the azimuth axis. Similar to the analysis of the two-position scheme in the previous text, the equivalent eastward gyro drift caused by the horizontal gyro in the two-position alignment process is:

(12) $$\varepsilon_{\omega E}=\frac{\int_{0}^{t}\left(\varepsilon_{x}\cos\omega t-\varepsilon_{y}\sin\omega t\right)dt}{t}$$

The estimation accuracy of azimuth error caused by horizontal gyro drift is:

(13) $$\delta {\hat{\phi }}_{U}^{\varepsilon }=-\frac{\int_{0}^{t}\left(\varepsilon_{x}\cos\omega t-\varepsilon_{y}\sin\omega t\right)dt}{t\omega_{ie}\cos L}$$

**Figure 3:**
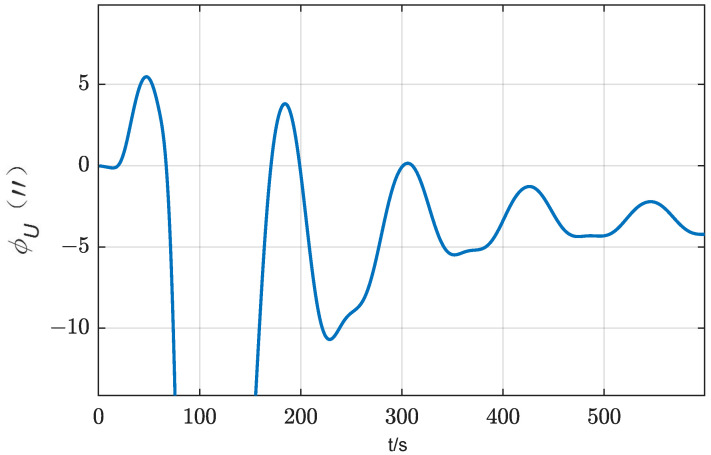
Influence of horizontal gyro bias on azimuth error (rotation scheme).

Observing the integral term in the Equation (13), it can be observed that when the IMU rotates around the *z*-axis for the entire cycle, its sine and cosine integral results are zero, indicating that the bias of the horizontal gyro is basically completely suppressed when rotated for one cycle. To verify the theoretical analysis, a simple simulation is conducted. Assume that the constant drift of horizontal axis gyro is 0.01°/h, and ignore other component errors and coarse alignment errors. The initial attitude is [0°, 0°, 0°]. IMU undergoes reciprocating rotational motion around the *z*-axis with an angular velocity of 6 °/s. The simulation results are shown in Figure 3.

From Figure 3, it can be seen that the bias of the horizontal gyroscope has been well modulated.

#### 3.1.2. Scale Factor Error

Because the alignment method analyzed in this paper includes the angular motion of IMU, the scale factor error cannot be ignored when some components with relatively poor scaling factor performance such as FOG [23] are used. Next, the influence of scale factor error during alignment is analyzed.

According to the attitude error equation, the attitude error introduced by the gyro scale factor error is:

(14) $${\dot{\phi }}_{K}=C_{b}^{n}C_{p}^{b}[\delta K_{g}]\omega_{ip}^{p}$$

where $C_{b}^{n}$ is the attitude transfer matrix, and $\omega_{ip}^{p}$ is gyroscopic output angular velocity. In the two-position alignment scheme, assuming that the body frame coincides with the navigation frame, take IMU’s positive rotation around the azimuth axis as an example, in Equation (15):

(15) $$C_{p}^{b}=\left[\begin{array}{ccc} \cos\omega t_{s} & -\sin\omega t_{s} & 0\\ \sin\omega t_{s} & \cos\omega t_{s} & 0\\ 0 & 0 & 1 \end{array}\right]$$

where $t_{s}$ is the rotation time of IMU, and ω is the rotation angular velocity of IMU. The attitude error caused by the gyro scale factor error can be obtained as:

(16) $$\int_{0}^{t}{\dot{\phi }}_{k}dt=\left[\begin{array}{c} \frac{\omega_{ie}\cos L\sin^{2}\omega t}{2\omega }\left(\delta K_{gx}-\delta K_{gy}\right)\\ \frac{\omega_{ie}\cos L\sin^{2}\omega t}{4\omega }\left(\delta K_{gy}-\delta K_{gx}\right)+\frac{t\omega_{ie}\cos L}{2}\left(\delta K_{gx}+\delta K_{gy}\right)\\ \delta K_{gz}\left(\omega_{ie}\sin L+\omega \right)t \end{array}\right]$$

Ignoring small quantities related to the rotation of the earth, during IMU rotation along the azimuth axis, the azimuth error caused by the gyro scale factor error can be simplified as:

(17) $$\phi_{KU}=\delta K_{gz}\omega t$$

**Figure 4:**
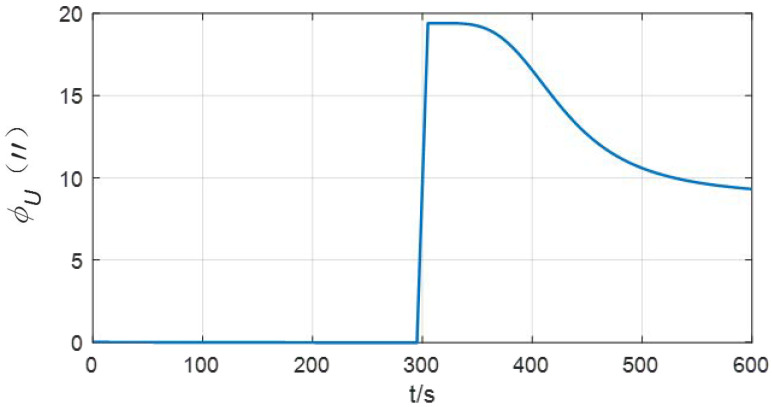
Influence of the scale factor error of azimuth gyro bias on the azimuth error (two-position scheme).

According to Equation (17), IMU rotation will cause azimuth error by coupling with the gyro scale factor error. When $\delta K_{gz}=30\;\mathrm{ppm}$, the attitude error caused by IMU rotation 180° is about 10″, as shown in Figure 4, which cannot be ignored. Therefore, for gyroscopes with unsatisfactory scale coefficient error, the effect of scale coefficient error of rotating axis gyroscopes should be taken into account when selecting the method of rotating IMU to improve the alignment accuracy. According to Equation (17), to suppress the influence of this error, the IMU needs to rotate the same angle in the opposite direction around the same rotation axis.

For the rotation scheme, when the IMU adopts the alignment scheme of a full cycle reciprocating rotation, according to Equation (17), the azimuth errors caused by scale factor error in the forward and reverse directions are as follows:

(18) $$\left\{\begin{array}{l} \phi_{KU+}=\delta K_{gz}\omega t\\ \phi_{KU-}=-\delta K_{gz}\omega t \end{array}\right.$$

**Figure 5:**
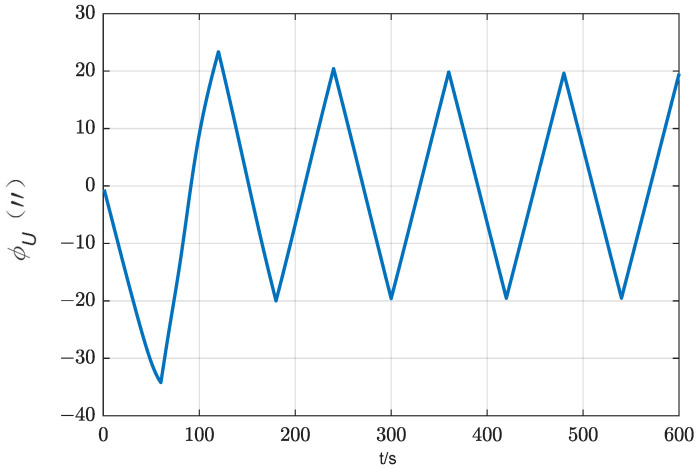
Influence of the scale factor error of azimuth gyro bias on the azimuth error (rotation scheme).

In this case, the estimation curve of the azimuth error will fluctuate up and down. Taking $\delta K_{gz}=30\;\mathrm{ppm}$ as an example, when rotating for one cycle, the fluctuation of the azimuth error is 30 ppm × 360° = 38.88″. When the IMU rotation direction changes, the direction of error accumulation also changes. The simulation results are shown in Figure 5.

#### 3.1.3. Misalignment

When the IMU rotates, the attitude error introduced by the misalignment of gyros is:

(19) $$\begin{array}{ll} {\dot{\phi }}_{G} & =C_{b}^{n}C_{p}^{b}G\omega_{ip}^{p}\\ & =\left[\begin{array}{c} \omega_{ie}\cos L\left(G_{xy}\cos^{2}\omega t-G_{yx}\sin^{2}\omega t\right)+\left(\omega_{ie}\sin L+\omega \right)\left(G_{xz}\cos\omega t-G_{yz}\sin\omega t\right)\\ \omega_{ie}\cos L\sin2\omega t\left(G_{xy}+G_{yx}\right)/2+\left(\omega_{ie}\sin L+\omega \right)\left(G_{xz}\sin\omega t+G_{yz}\cos\omega t\right)\\ G_{zx}\omega_{ie}\cos L\sin\omega t+G_{zy}\omega_{ie}\cos L\cos\omega t \end{array}\right] \end{array}$$

When the IMU rotates in a positive direction around the azimuth axis, it can be obtained when the small amount related to the Earth’s rotation is ignored ($\phi_{GU}=0$). When the IMU rotates around the azimuth axis, the misalignment of the gyro will not affect the azimuth alignment accuracy. For horizontal attitude alignment accuracy, IMU rotation will also introduce corresponding errors, as shown in Equation (20):

(20) $$\left\{\begin{array}{c} \phi_{GE}=-G_{xz}\sin\omega t-G_{yz}\cos\omega t\\ \phi_{GN}=G_{xz}\cos\omega t-G_{yz}\sin\omega t \end{array}\right.$$

**Figure 6:**
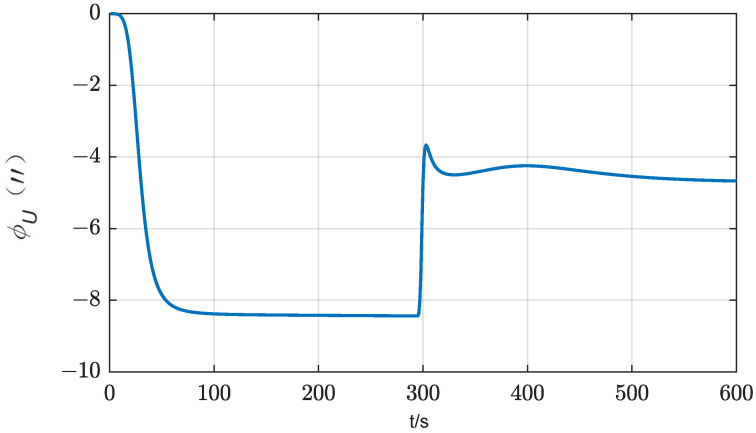
Influence of the misalignment of the azimuth gyro on the azimuth error (two-position scheme).

The simple simulation analysis is carried out with 5″ misalignment of the gyro, and the results are shown in Figure 6. According to the simulation results, the size of the error is basically the same level as the misalignment of the gyro. The reason why the heading error in the simulation is not zero is that the influence of the Earth’s rotational velocity was ignored in the derivation. Under the premise that the calibration accuracy of the misalignment is high, the importance of this error could be ranked after the scale factor error.

**Figure 7:**
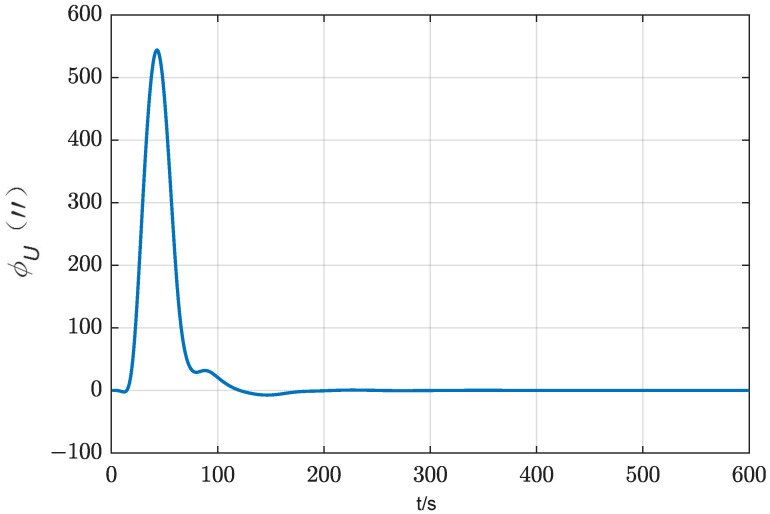
Influence of the misalignment of the azimuth gyro on the azimuth error (rotation scheme).

For the rotation scheme, the simulation results are shown in the Figure 7.

Owing to the full rotation of the scheme, the misalignment of the azimuth gyro has also been modulated.

To sum up, the main gyro error items that affect the alignment accuracy include the constant bias of the horizontal gyro, the gyro scale factor error of the rotation axis, and the gyro misalignment related to the rotation axis. Among them, the constant bias is the main influence factor. The gyro scale factor error only introduces the relevant alignment error when the IMU is rotating, and the effect of gyro misalignment is the smallest.

### 3.2. Alignment Scheme Design

#### 3.2.1. Alignment and Rotation Scheme Design

**Figure 8:**
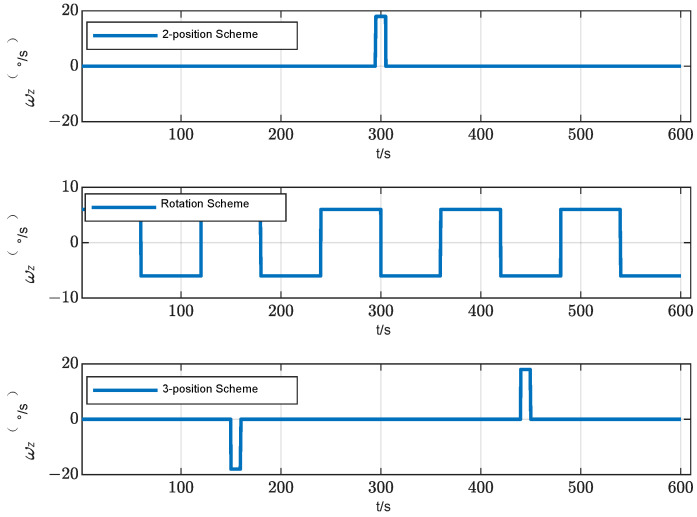
Schematic diagram of three alignment schemes.

According to the theoretical analysis, the two-position alignment scheme only considers the effect of gyro constant bias on the alignment accuracy, and ignores the alignment error introduced by other related error items when IMU rotates. Therefore, when designing the multi-position alignment scheme, it is necessary to comprehensively consider the various errors of the components and the modulation effect of the rotation scheme on the various errors. According to the theoretical analysis in Section 2.1 and Section 2.2, the main factors that affect the initial alignment accuracy are the constant bias and the scale factor error of the gyro. To suppress the effect of constant bias, the IMU needs to remain at the same time in the symmetrical position, while to suppress the effect of the gyro scale factor error, the IMU needs to turn the same angle around the positive and negative directions of the rotation axis. Therefore, in this study, we designed a three-position alignment scheme that rotates twice around the azimuth axis. In this scheme, the IMU rotates 180° forward and backward around the azimuth axis to ensure the suppression effect of the gyro scale factor error. It stays at the first position and the third position for the same time, and stays at the second position for twice as long as at the first position to ensure the suppression effect on the constant bias of the gyro. During the alignment process, the *z*-axis gyro output of the two-position scheme, rotation scheme and three-position schemes is shown in Figure 8.

#### 3.2.2. Alignment Algorithm Design

In the traditional initial alignment scheme, the initial alignment algorithm uses a 12-dimensional state vector Kalman filter for alignment solution. In this paper, due to the introduction of the research on the influence of the gyroscopic scale factor error of the rotating axis on the alignment accuracy, the gyro scale factor error of the rotating axis is introduced into the state vector of the initial alignment filter, namely:

(21) $$X_{A}={\left[\begin{array}{llllllllllll} \varphi_{E} & \varphi_{N} & \varphi_{U} & \delta V_{E} & \delta V_{N} & \delta V_{U} & \varepsilon_{x}^{p} & \varepsilon_{y}^{p} & \varepsilon_{z}^{p} & \nabla_{x}^{p} & \nabla_{y}^{p} & \nabla_{z}^{p} \end{array}\delta K_{gz}\right]}^{T}$$

The state vectors in Equation (21) are attitude error, velocity error, gyroscope bias, accelerometer bias and scale factor error of the *z*-axis gyroscope.

The state equation and measurement equation are [24,25]:

(22) $$\dot{X}=FX+W$$

(23) Z=HX+V

where ***W*** and ***V*** represent the state noise matrix and the measurement noise matrix, respectively. The velocity output calculated by the system is selected as the measurement information. According to the IMU error model and the INS error model, the state transition matrix in the filter can be obtained as:

(24) $$F={\left[\begin{array}{ccc} F_{1} & F_{2} & F_{3}\\ 0_{7\times 6} & 0_{7\times 6} & 0_{7\times 1} \end{array}\right]}_{13\times 13}$$

In Equation (24), each sub-matrix is:

(25) $$F_{1}=\left[\begin{array}{cccccc} 0 & \omega_{ie}\sin L & -\omega_{ie}\cos L & 0 & -\frac{1}{R_{M}+h} & 0\\ -\omega_{ie}\sin L & 0 & 0 & \frac{1}{R_{N}+h} & 0 & 0\\ \omega_{ie}\cos L & 0 & 0 & \frac{\tan L}{R_{N}+h} & 0 & 0\\ 0 & -f_{U} & -f_{U} & 0 & 2\omega_{ie}\sin L & -2\omega_{ie}\cos L\\ f_{U} & 0 & -f_{E} & -2\omega_{ie}\sin L & 0 & 0\\ -f_{N} & f_{E} & 0 & 2\omega_{ie}\cos L & 0 & 0 \end{array}\right]$$

(26) $$F_{2}=\left[\begin{array}{cc} -C_{p}^{n} & 0_{3\times 3}\\ 0_{3\times 3} & C_{p}^{n} \end{array}\right]$$

(27) $$F_{3}=-{\left[\begin{array}{ll} C_{p}^{n}{\left[\begin{array}{lll} 0 & 0 & \omega_{ipz}^{p} \end{array}\right]}^{T} & 0_{1\times 3} \end{array}\right]}^{T}$$

The measurement matrix can be represented as $H=[\begin{array}{lll} 0_{3\times 3} & I_{3\times 3} & 0_{3\times 7} \end{array}]$; $I_{3\times 3}$ represents a third-order identity matrix, and $0_{m\times n}$ represents an *m* × *n* zero matrix.

## 4. Simulation and Experimental Analysis

### 4.1. Simulation Analysis

**Figure 9:**
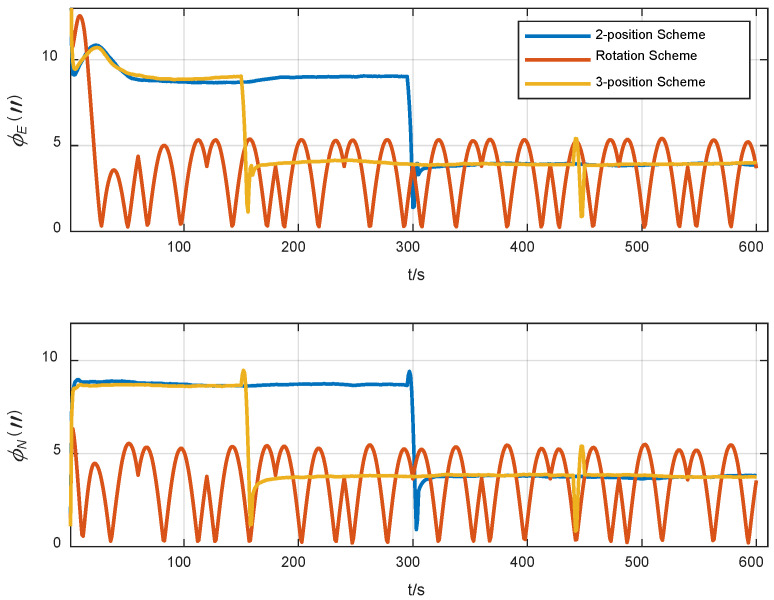
Curves of the horizontal attitude error of three alignment schemes.

**Figure 10:**
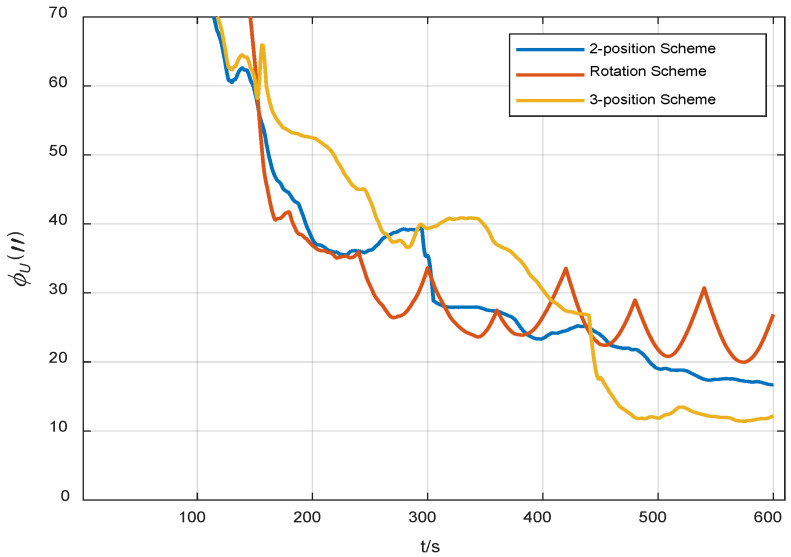
Curves of the azimuth error of three alignment schemes.

In order to evaluate the alignment accuracy of the three-position alignment scheme, a comparative simulation experiment was carried out for three alignment schemes: two-position alignment, rotation alignment and three-position alignment. Assume that the initial attitude error is [0.5°, 0.5° 1°] and the position is [108.9° E, 34.2° N, 400 m], and the alignment time is 600 s. IMU error parameter settings in the simulation are shown in Table 1. In total, 20 simulation tests on the three alignment schemes are carried out, and the root mean square curves of the system attitude error during the alignment process are drawn, as shown in Figure 9 and Figure 10.

It can be seen from Figure 9 and Figure 10 that the horizontal attitude error converges rapidly as the IMU rotates in the two-position scheme and three-position scheme. The reason is that the horizontal attitude alignment accuracy is mainly affected by the horizontal equivalent accelerometer bias. As the accelerometer bias is rapidly and accurately estimated, the horizontal attitude error converges rapidly. In the rotation alignment scheme, due to the continuous rotation of IMU, it can be seen from the analysis in Section 3.1.3 that although the horizontal accelerometer bias can be accurately estimated and compensated, the gyro misalignment will cause the horizontal attitude to be sinusoidal. The misalignment of the gyro is also the reason why the horizontal error curve of the two-position scheme and the three-position scheme cannot be further converged. However, in terms of the magnitude of the alignment error caused by misalignment of the gyro, its effect is not as obvious as that of gyro constant bias and gyro scale factor error.

As can be seen from Figure 10, from the final result, the azimuth alignment accuracy of the three-position alignment scheme is similar to that of the rotation alignment scheme, which is better than the two-position alignment scheme. Among them, the azimuth error curve of the two-position alignment scheme fluctuates obviously when the alignment time is about 300 s. According to Section 3.1.2, this is caused by the coupling of the gyro scale factor error and IMU rotation motion, which is consistent with the fluctuation of error curve of the three-position scheme around 180 s and 440 s. But the two rotation directions of the three-position alignment scheme are opposite, which effectively suppresses the influence of the gyro scale coefficient error on the alignment accuracy. The continuous fluctuation of the angular error curve of the rotation alignment scheme is caused by the continuous rotation of the IMU. Since the gyro scale factor error of the rotating axis cannot be accurately estimated and compensated, the continuous rotation of IMU will cause continuous fluctuation of azimuth.

The azimuth accuracy of the rotation alignment scheme at the end of alignment is similar to that of the three-position alignment scheme. However, in some special applications that require a rapid response, such as self-propelled guns, missile launchers, etc., the rotation alignment scheme may encounter some problems. In such cases, the vehicle may be temporarily transferred before the preset alignment time. In the circumstances that the IMU is not at the end of a complete cycle (forward and reverse for one cycle), the adoption of the rotating alignment scheme means that the scale factor error of the gyroscope is not fully modulated, so the alignment results obtained at this time are not ideal. It can also be seen from the azimuth error angle curve of rotation alignment in Figure 10 that the scheme fluctuates greatly during the alignment process. Once the alignment needs to be temporarily terminated in advance, the credibility of the obtained alignment results will be greatly reduced.

**Figure 11:**
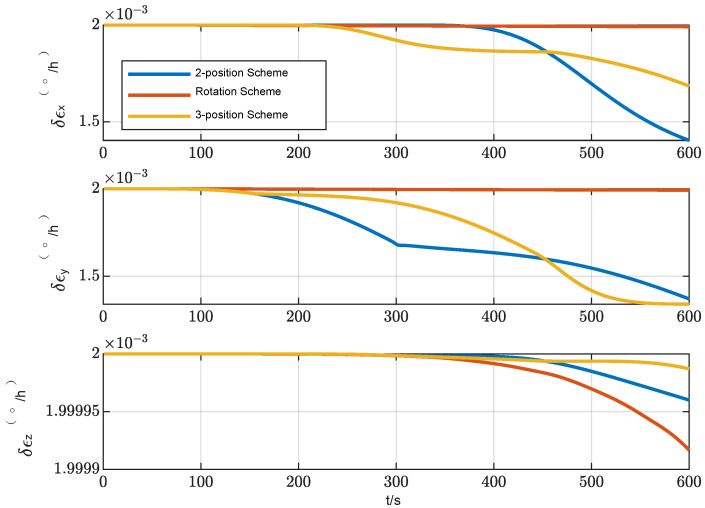
Standard deviation of the estimation error of gyro bias.

**Figure 12:**
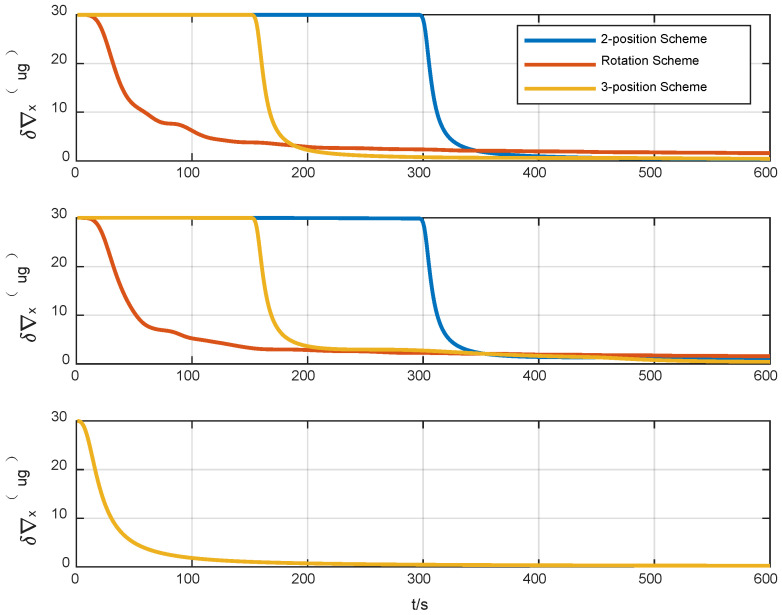
Standard deviation of the estimation error of accelerometer bias.

**Figure 13:**
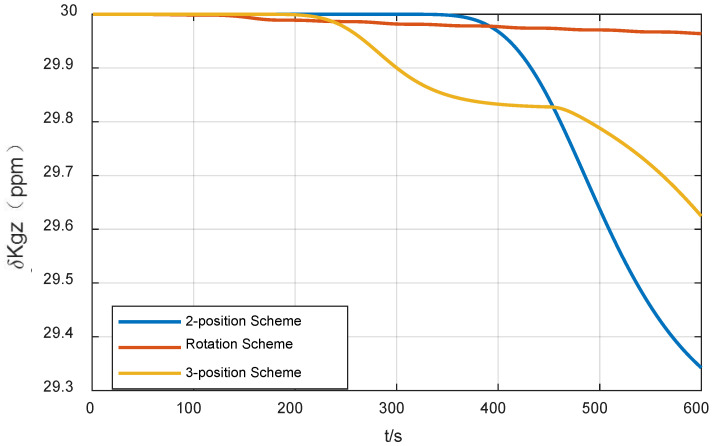
Standard deviation of the estimation error of the scale factor error of *Z*-axis gyro.

A statistical analysis of the standard deviation of the estimation error was conducted. From Figure 11, Figure 12 and Figure 13, it can be seen that the standard deviation curve of the estimation error of accelerometer bias can converge in the vicinity of zero, indicating that the filter has a good estimation effect on accelerometer bias, while the filter has poor estimation effect on gyro bias and *z*-axis gyro scale factor error. For gyro bias, these schemes all adopt the idea of error modulation to suppress their influence. Due to the inability to estimate the scale factor error of the *z*-axis gyroscope, the two-position scheme generates additional errors. These analyses are also consistent with the results of the analysis in Section 3.1.

### 4.2. Test Verification

**Figure 14:**
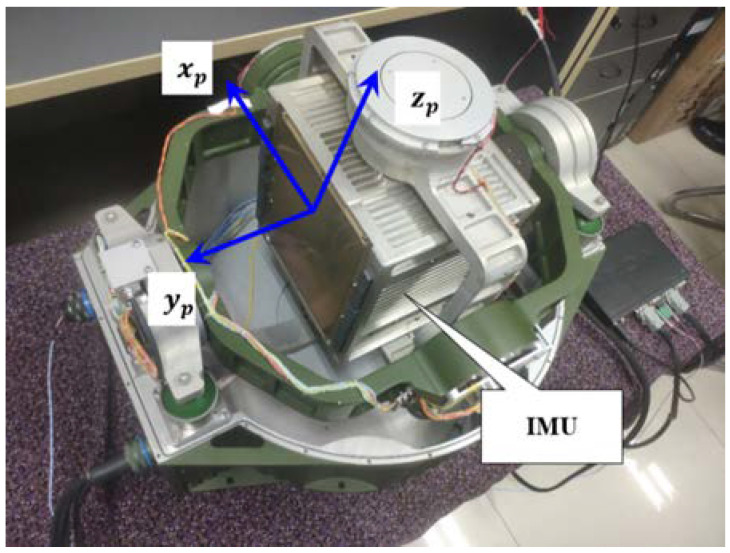
Rotational inertial navigation system (RINS) for the experiment.

In order to verify the research content of this paper, the two-position alignment, rotation alignment and three-position alignment schemes were compared and analyzed through experiments. The experimental equipment mainly includes a three-axis rotary fiber optic gyro inertial navigation system, as shown in Figure 14. Inertial component parameters adopted by the system are basically consistent with the simulation parameters. Considering the high accuracy of the system and the difficulty in obtaining the high-precision attitude reference information matched with it, ten experiments were carried out on three alignment schemes respectively, then the standard deviations of the alignment results were calculated, and the statistical results were compared and analyzed. The results are shown in Table 2.

**Figure 15:**
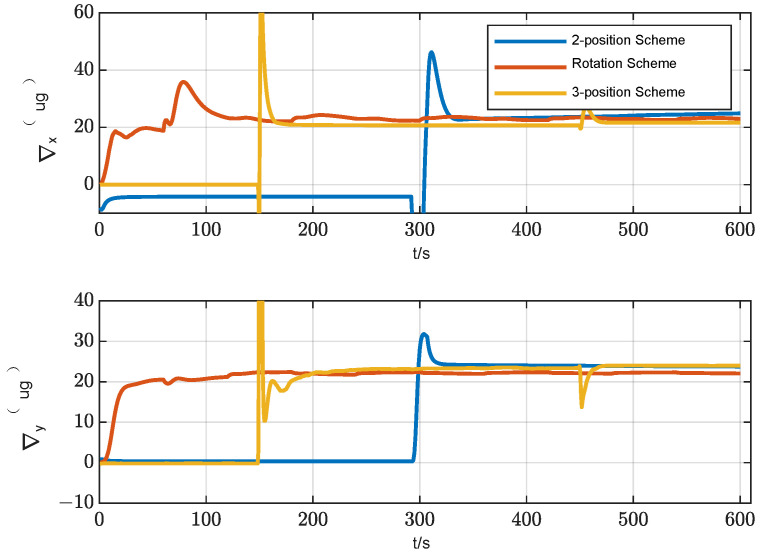
Estimation curve of horizontal accelerometer bias.

**Figure 16:**
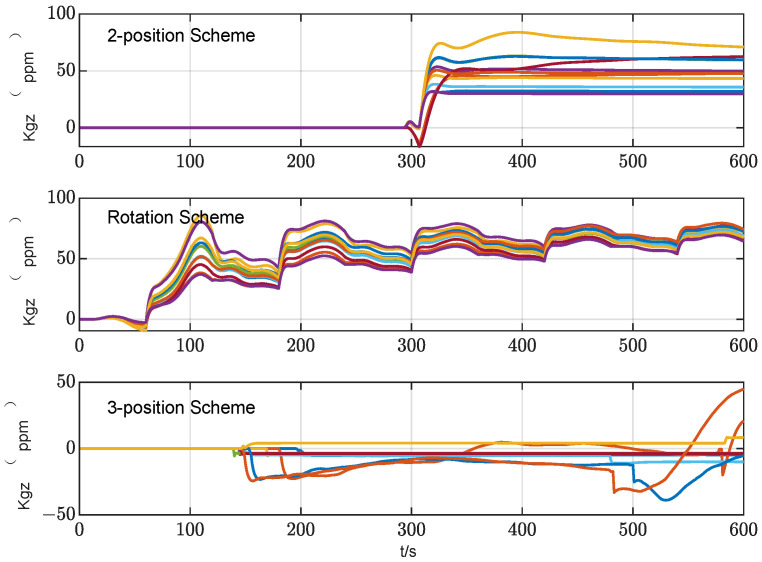
Estimation curve of gyroscope scale factor error of the rotating axis.

It can be seen from Table 2 that the horizontal alignment accuracy of the three alignment schemes is basically the same. The reason is that the three alignment schemes can accurately estimate the bias of the horizontal accelerometer, which is the main error that affects the accuracy of horizontal alignment. The estimation result of the bias of the horizontal accelerometer is shown in Figure 15. From the perspective of azimuth alignment accuracy, the three-position alignment scheme designed in this paper can reach the same accuracy level as the rotation alignment scheme, which is significantly improved compared with the two-position alignment scheme. According to the analysis in Section 2 of this paper, the reason is that in the three-position alignment scheme, the equivalent biases of the horizontal gyros in the second position and the first and third positions are modulated. The two-position scheme and the rotation scheme can also achieve the corresponding error modulation. However, the three-position scheme suppresses the influence of the gyro scale factor error on azimuth alignment accuracy through two angular movements with opposite directions and the same rotation angle, while the two-position alignment scheme only rotates once, and the azimuth error introduced by the gyro scale factor error cannot be modulated. In the rotation alignment scheme, the coupling between the continuous rotation of IMU and the error of the gyro scale factor causes the continuous fluctuation of the azimuth error. The above three schemes cannot estimate the gyro scale factor error of the rotating axis, as shown in Figure 16. Therefore, the influence of this error term can only be suppressed by error modulation. The above experimental results are basically consistent with the theoretical analysis and simulation results of this paper, which verifies the effectiveness of the alignment error suppression theory and the alignment scheme designed in this paper.

## 5. Conclusions

This paper investigates the influencing factors and principles for error suppression in multi-position initial alignment accuracy. Using the classic two-position alignment scheme, an analysis is conducted on the primary component error terms and their respective magnitudes impacting alignment accuracy during IMU rotation. Notably, the bias of gyroscopes and the gyro scale factor error emerge as principal factors influencing alignment accuracy within the two-position alignment paradigm. Building upon an elucidation of the suppression principle of IMU rotation on initial alignment errors, a novel three-position initial alignment scheme is proposed, accompanied by a detailed analysis of its error suppression mechanism. Simulation and experimental outcomes corroborate the efficacy of the proposed scheme in effectively mitigating the adverse effects of gyro scale factor error and gyro bias on initial alignment accuracy. Furthermore, the error suppression strategies outlined herein offer valuable insights for the design of initial alignment schemes tailored to diverse devices operating under corresponding conditions.

## Figures and Tables

**Table 1:** IMU error parameter settings in the simulation.

Error Terms		Parameters
Gyro	Constant bias	0.002°/h
	Random walk	0.0003°/√h
	Scale factor error	30 ppm
	Misalignment	2″
	Driving noise (first-order Markov process)	0.0003°/h
	Correlated time	3000 s
Accelerometer	Constant bias	20 μg
	Random walk	5 μg/√Hz
	Scale factor error	30 ppm
	Misalignment	2″

**Table 2:** Standard deviation statistics of the alignment results of three alignment schemes.

Attitude Angle	Two-Position Scheme	Rotation Scheme	Three-Position Scheme
pitch	4.59″	2.19″	3.51″
roll	1.32″	1.58″	2.03″
yaw	20.32″	14.63″	12.83″

## Data Availability

Data is contained within the article.
